# Is Household Air Pollution a Risk Factor for Eye Disease?

**DOI:** 10.3390/ijerph10115378

**Published:** 2013-10-25

**Authors:** Sheila K. West, Michael N. Bates, Jennifer S. Lee, Debra A. Schaumberg, David J. Lee, Heather Adair-Rohani, Dong Feng Chen, Houmam Araj

**Affiliations:** 1 Wilmer Eye Institute, Johns Hopkins Hospital, Baltimore, MD 21287, USA; E-Mail: jenleesf@gmail.com; 2 School of Public Health, Divisions of Epidemiology and Environmental Health Sciences, University of California, Berkeley, CA 94720, USA; E-Mail: m_bates@berkeley.edu; 3 John A. Moran Eye Center, Department of Ophthalmology and Visual Sciences, University of Utah School of Medicine, Salt lake City, UT 84132, USA; E-Mail: debra.schaumberg@utah.edu; 4 Department of Epidemiology, Harvard School of Public Health, Boston, MA 02215, USA; 5 Schepens Eye Research Institute, Massachusetts Eye and Ear, Department of Ophthalmology, Harvard Medical School, Boston, MA 02114, USA; E-Mail: dongfeng_chen@meei.harvard.edu; 6 Department of Epidemiology and Public Health, University of Miami Miller School of Medicine, Miami, FL 33101, USA; E-Mail: dlee@med.miami.edu; 7 World Health Organization, Geneva CH-1211, Switzerland; E-Mail: hradair@gmail.com; 8 National Eye Institute, Bethesda, MD 20892, USA; E-Mail: arajh@mail.nih.gov

**Keywords:** biomass, blindness, cataract, trachoma, dry eye disease, household air pollution

## Abstract

In developing countries, household air pollution (HAP) resulting from the inefficient burning of coal and biomass (wood, charcoal, animal dung and crop residues) for cooking and heating has been linked to a number of negative health outcomes, mostly notably respiratory diseases and cancers. While ocular irritation has been associated with HAP, there are sparse data on adverse ocular outcomes that may result from acute and chronic exposures. We consider that there is suggestive evidence, and biological plausibility, to hypothesize that HAP is associated with some of the major blinding, and painful, eye conditions seen worldwide. Further research on this environmental risk factor for eye diseases is warranted.

## 1. Introduction

According to the World Health Organization (WHO), 285 million people worldwide are visually impaired, including 39 million who are blind [1]. Furthermore, 90% of all blind individuals live in developing countries [2]. The major causes of blindness worldwide are cataract (~50%), glaucoma (~15%), corneal opacities (~10%), and trachoma (~6%), [2]. Trachoma is the leading infectious cause of blindness worldwide. Women are more likely to be blind than men, and to suffer disproportionately from the major causes of eye disease, cataract and trachoma.

Countries in the developing world are also the main users of solid fuels (biomass and coal) for cooking and heating. Approximately three billion people worldwide—nearly half the World’s population—including almost all in the developing World, are users of solid fuel [3]. Biomass consists primarily of wood, charcoal, crop residues, and animal dung. These fuels, often burned in “three-stone cooking fires” (three stones in a triangle with a fire in the center) or simple traditional stoves without chimneys, are inefficient and highly polluting, leading to high concentrations of particulate matter, carbon monoxide and other organic compounds [4]. For example, emissions from charcoal have been found to include mercury and trace metals as well as aromatic organic compounds [5,6,7].

In addition, the food itself that is cooked, either fried or grilled at high temperature, can emit smoke and volatile organic compounds [8]. For instance, use of unrefined cooking oils, under high temperature cooking conditions, has been shown to release toxic compounds such as benzene and formaldehyde [9]. Women, who are typically responsible for cooking in these households, are the most exposed to these pollutants, along with their young children.

There is scarce research into any association of eye diseases with exposures to cooking with solid fuels, although good evidence exists linking the inefficient use of these fuels to a wide range of health effects, including pneumonia, cancer, chronic obstructive pulmonary disease, low birthweight and cardiovascular disease [10]. Household Air Pollution (HAP) from solid fuel use is the third leading risk factor for the global burden of disease, ranking only after high blood pressure and tobacco smoking [11]. The respiratory diseases are thought to result from the inhalation of toxic compounds [4]. Furthermore, research suggests that inhalation of compounds emitted from the incomplete combustion of solid fuels can result in sufficient systemic levels that lead to cardiovascular disease in exposed individuals [12] and low birthweight in children born to exposed women [13,14,15].

Many of the studies regarding hazards of HAP have reported “ocular irritation” as a complaint, and indeed it appears to be a consistent finding when symptoms are elicited [16,17,18,19]. In a study of cookstoves in Guatemala, more than 60% of women at baseline reported their eyes were always irritated. There was a substantial reduction in prevalence of these symptoms among the women randomized to the less polluting (chimney) stoves (odds ratio [OR] 0.18, 95% confidence interval [CI] 0.11–0.29), suggesting that the symptoms may be reversible once the offending stimulus is removed [16]. Similar results came from an intervention in which some Pakistani women received a clay chimney stove [18]. The OR for “tears while cooking” (TWC) for the chimney stove relative to the traditional 3-stone stove was 0.51 (95% [CI] 0.21–1.21). TWC assessment has also been suggested as an inexpensive HAP exposure indicator when exposure-measuring equipment is not available [17]. However, there has been no systematic attempt to date to determine if HAP is associated with true ocular disease. One recent review of diseases associated with HAP due to the use of biomass fuels, did include cataract, based on one study, and suggested a link with biomass smoke condensates [20]. However, the absence of studies investigating a link with other eye diseases does not mean there is no relationship, and the need for research in this area is compelling.

The co-occurrence in developing countries of exposure to household fuel combustion products and higher rates of blindness, especially in females [21], makes it imperative that any causal relationships between the two be better understood.

The purpose of this paper is to summarize the evidence to date for a relationship between household air pollution and eye disease and to determine if there are ocular conditions that have not been studied but which have biological plausibility for an association with HAP. Additionally, we outline needs for future research on the relationship between household fuel use and these eye diseases.

## 2. Methods

The authors conducted a methodical review of the literature. The key inclusion criterion for this review was that the publication must have reported data from a study of ocular disorders in relation to household fuel use in a low- or middle-income country, with relative risk estimates adjusted for, at minimum, age and sex. We first searched PubMed for eligible studies using the following keywords for ocular impairment: “cataract”, “trachoma”, “dry eye”, “macular degeneration”, “blindness”, “tears” and “eye disease”, each in combination with the following exposure terms: “indoor air pollution”, “household air pollution”, “biomass”, “cookstove”, “cooking”, “smoke”, “stove” and “fuel”. The search was updated on 12 July 2013, at which time it identified 205 publications. Many of these were studies of tobacco smoke, which were not relevant to this review.

After reviewing abstracts and peer-reviewed publications, 12 relevant publications were identified from the PubMed results and critically reviewed. An identical search strategy was applied to Google Scholar, identifying a further six publications. Although reference lists of identified publications were searched, no further publications were identified from these. All publications were in English, although one was in Indonesian, with an English abstract.

We identified a total of 18 relevant study publications, documenting 19 studies. Eleven studies were of cataract, four of trachoma, and two generic studies of blindness or visual impairment. The last two publications described symptoms of tears while cooking, and eye irritation.

## 3. Results

Generally, in the literature linking eye disease and HAP, the characterization of exposure to HAP was not well described (Table 1). The types of household fuel used and the devices in which it was burned were often not presented. Some inferences can nevertheless be made: for example, in the studies on trachoma in Tanzania, the geographical location suggests that all cooking fires were simple pits, and wood or charcoal was used as fuel [22,23]. In other studies, stoves with and without chimneys/flues were not separately categorized, and kitchen ventilation was usually not considered in the statistical analysis. There is evidence that these factors may be important for understanding the relationship to chronic eye disease, like cataract [24].

Another issue with these studies was the analyses of the data, which often combined different fuel types into single categories. For example, kerosene, which can be a highly polluting fuel [25], was sometimes included with biomass and other times included with the “clean fuel” reference group. Depending on whether and how kerosene combustion products affect the eye (about which there is little information), the decision as to the group in which to include kerosene may have raised or lowered relative risk estimates for biomass fuel use.

Finally, most studies relied solely on assessing the current stove and fuel type. In many parts of Africa, this is a reasonable surrogate for long-term exposure. However, cataract, scarring trachoma, and ocular surface disease may well be the result of long-term exposures and as such, either longitudinal studies or the collection of a more complete history of past exposures would be preferable. We summarize the findings of the review below.

### 3.1. Eye Diseases Related to Smoking

#### 3.1.1. Cataract

Cataract has long been linked to cigarette smoking, and, according to the United States Surgeon General, there is sufficient evidence to label smoking as a causal factor for cataract [26]. This points to a mechanism for inhaled toxic substances that either directly or indirectly adversely affect lens tissues. While it is not possible to identify the exact compound(s) in cigarette smoke that are responsible for the lens toxicity, there are a number of plausible candidates, such as naphthalene, that are also found in biomass fuels [27]. Napthalene is well known for its cataractogeneic potential and is indeed used to induce cataract in animal models [28]. Of note, biomass smoke condensates also contain metal ions such as lead, and lead exposure is associated with protein aggregation diseases like cataract [7,29]. Thus, there is reasonable plausibility to hypothesize a linkage between cataract and HAP exposure.

In support of this hypothesis, the 11 studies identified to date suggest an association between biomass fuel use and cataract, with relative risk estimates almost all greater than 1.0 (Table 1) [24,30,31,32,33,34,35,36,37,38]. However, there are issues with this literature. Most of the investigations of biomass fuel and cataract have been case-control studies, and their validity relies heavily on the proper selection of controls. One study used controls diagnosed with refractive errors [24]; however, refractive error is also associated with more education, and those with more education have lower cataract risk [39]. Thus, the possibility of selection bias cannot be ruled out. Most case-control studies were also hospital or clinic based, and control selection procedures were not well-described, so the likelihood of selection bias could not be evaluated.

An additional consideration is the possibility that high ambient temperature is associated with cataract by denaturing lens proteins [40]. It is possible that chronic heat exposure is a mechanism by which household cooking and heating fires could induce cataract, although this would be difficult to distinguish from a HAP-induced effect.

Another limitation is that most studies did not investigate the relationship with cataract subtypes (nuclear, posterior sub-capsular and cortical). This distinction may be important since, for example, cigarette smoking predominantly causes nuclear cataract. If HAP acts similarly, combining cataract subtypes may have attenuated effect estimates. Finally, most studies of the relationship with cataract were conducted in India, and these findings may not be more widely generalizable.

In countries where HAP exposure is high, it would be valuable to study the relationship with cataract, while simultaneously using accepted approaches to grade the different subtypes of lens opacity and paying careful attention to selection of control populations.

#### 3.1.2. Age Related Macular Degeneration (AMD)

AMD is another chronic eye disease that is related to smoking, and there is also modest evidence for a relationship with secondhand tobacco smoke [41,42,43]. AMD is most common in high-income countries, and there is a lower prevalence of the disease in Latinos and African Americans compared to Caucasians [44,45,46,47]. It is not well studied in populations in less developed countries and we identified no studies that investigated whether AMD was associated with household solid fuel use.

As life expectancies increase in many countries, the association (if any) between HAP and AMD may be a reasonable avenue for future research. There are experimental data to posit a potential relationship based on the association with smoking. Others have found that experimental exposure to carbon monoxide at 500 ppm causes an increase in arterial and venous diameters, retinal blood flow, and other measures of ocular blood flow [48].

Of note, the main genetic polymorphisms associated with severe AMD are those that regulate innate immunity and inflammation, and consequently dysregulation of the immune system is an active area of research into the etiology of AMD [49,50]. Epidemiologic studies have shown that exposure to fine particles associated with solid fuel use causes increased production of inflammatory cells and elicits systemic inflammatory responses associated with increased cytokine production [10,51,52,53]. One could speculate that a mechanism of action for HAP may be chronic exacerbation of the immune response, in conjunction with genetic AMD-risk variants, which may result in degeneration of retinal cells and neovascularization.

#### 3.1.3. Dry Eye Disease (DED)

Dry eye disease, while not a major cause of blindness, is associated with substantial ocular pain and discomfort and can lead to fluctuating visual disturbances [54,55]. It is a common eye condition, and is prevalent in many developing countries [56].

**Table 1:** Summary of relevant literature for household fuels and cataract, trachoma and other vision disorders.

Reference	Study design ^1^	Location	Study population ^2^	Exposure measure	RR (95% CI)	Adjusted for:	Strengths	Limitations
*Outcome: Cataract*								
Mohan *et al*. (1989) [31]	CC	India (New Delhi)	1,441/549 Both sexes, 37–62 years.	Cow dung and wood *vs*. gas.	1.61 (1.02–2.50) (C, N & M ^3^)	Age, sex, year of examination, aspirin, education, dietary protein, BP, BMI, cloud cover, time doing near work.	Large size; clinical confirmation.	Hospital-based. Controls not well-described. Unclear how kerosene treated as exposure (possibly as “gas”). Limited data on possible confounders provided.
Badrinath *et al*. (1996) [30]	CC	India (Chennai)	244/264 Both sexes, 40–60 years.	Cheap cooking fuel (cow dung, wood, coal, kerosene).	4.91 (2.80–8.50) Cooking fuels did not appear in the final multivariate model	Age, sex.	Clinical confirmation.	Hospital-based. Controls not well-described. Cataract types not distinguished. Cooking fuels not in final model, suggesting confounding of fuel type. Fuel types combined.
Ughade *et al*. (1998) [37]	CC	India (Nagpur)	262/262 Both sexes, no age restriction.	Cheaper cooking fuel (cow dung, wood, coal).	4.13 (2.66–6.40)	Low SES, illiteracy, history of diarrhea, diabetes, glaucoma, myopia, smoking history, hypertension.	Clinical confirmation.	Hospital-based. Controls not well-described. Cataract types not distinguished.
Zodpey & Ughade (1999) [38]	CC	India (Nagpur)	223/223 Females, 35–75 years.	Less expensive cooking fuels (cow dung, wood, coal, kerosene) *vs*. gas.	2.37 (1.44–4.13)	Age, SES.	Clinical confirmation.	Study population may overlap with Ughade (1998). Includes kerosene with biomass and coal. No fuel type breakdown provided. Controls not well described. Cataract types not distinguished. Limited data on possible confounders provided.
Sreenivas *et al*. (1999) [34]	CC	India (Angamally)	258/308 Both sexes, 40–60 years.	Wood, cow dung *vs*. others.	0.37 (0.02–6.65)	Age, sex.	Clinical confirmation.	Controls not well described. Cataract types not distinguished. “Other” fuels not described.
Sreenivas *et al*. (1999) [34]	CC	India (Calcutta)	301/591 Both sexes, 40–60 years.	Wood, cow dung *vs*. others.	2.06 (1.31–3.23)	Age, sex, sunlight exposure, BP, height, hours of work/day.	Clinical confirmation.	Controls not well described. Cataract types not distinguished. “Other” fuels not described.
Pokhrel *et al*. (2005) [24]	CC	Nepal, India	206/203 Women, 35–75 years.	Solid fuel *vs*. biogas, LPG and kerosene.	Vented stove: 1.23 (0.44–3.42) Unvented stove: 1.90 (1.00–3.61)	Age, kitchen ventilation, work outside, literacy, area of residence, source of light, incense burned.	Clinical confirmation. Detailed information on possible confounders. Exposure history, stove type, lighting and ventilation taken into account.	Visual acuity controls—possible selection bias. Inclusion of kerosene in comparison group.
Saha *et al*. (2005) [33]	CS	Western India	469 Both sexes.	Biomass, wood, kerosene, coal, LPG, considered separately and together.	Only biomass *vs*. only LPG: 2.40 (0.90–6.37) Only wood *vs*. only LPG: 3.47 (1.05–11.5)	Unclear, but presumed: age, sex, income, smoking, diabetes, hypertension, house type.	Clinical confirmation. Fuels considered separately.	Cataract types not distinguished. Limited data on possible confounders provided.
Tana *et al*. (2009) [35] (English abstract only)	CS	Indonesia	95,800 women.	Charcoal, wood, kerosene *vs*. gas.	Charcoal: 1.83 (1.43–2.34) Wood: 1.49 (1.31–1.68) Kerosene: 1.37 (1.22–1.54)	Not available.	Large study size.	Insufficient information to judge.
Tanchangya & Geater (2011) [36]	CC	Bangladesh	Females: 80/160 Males: 73/146 18–49 years.	Wood/dry leaves, rice straw, cow dung.	Females: Rice straw-used *vs*. never used: 1.95 (1.03–3.69) Cow dung-used *vs*. never used: 0.45 (0.24–0.84) Males: Wood/ dry leaves-used *vs*. never used: 1.84 (0.89–3.93)	Education level, family history of cataract, smoking status (males only).	Clinical confirmation. Fuels considered separately.	Cataract types not distinguished. Hospital-based.
Pokhrel *et al*. (2013) [32]	CS	Nepal (Pokhara)	143 women	Biomass and kerosene *vs*. gas	(all ORs) Nuclear opacity: Biomass 2.58 (1.22–5.46); Kerosene 5.18 (0.88–30.38) Nuclear color:Biomass 1.98 (0.82–4.74); Kerosene 5.48 (1.23–24.37) Cortical cataract: Biomass 0.68 (0.19–2.39); Kerosene 0.83 (0.12-5.55)	Heating fuel, kitchen ventilation, smoking, mosquito coil use, nutritional status, residence, land ownership, literacy, occupation, age, income	Clinical confirmation; cataract types evaluated separately; adjusted for multiple potential confounders	Small sample size for kerosene subgroup
*Outcome: Trachoma/Trichiasis*								
Taylor *et al*. (1989) [22]	CS	Tanzania	3,832 preschool children from 1,928 households.	Cooking fire in sleeping room.	Active trachoma: 1.24 (1.08–1.43)	Age, sex, unclean face, handkerchief use, towel use, traditional beliefs, fly score, cattle herding, >30 min to water source, latrine present.	Large sample; clinical confirmation.	Fuel type, stove not described. We can infer from the area in Tanzania that this was a straight fire, using wood or charcoal.
Sahlu & Larson (1992) [57]	CS	Ethiopia	1,222 people from 221 households. Both sexes, all ages.	Cooking in central room *vs*. in separate room.	Trachoma: 0.38 (0.31–0.45)	Age, sex, head of household characteristic and their education, altitude, garbage disposal distance from house, crowding, animal ownership, animals inside house.	Large sample; clinical confirmation.	Fuel type, stove not described. No adjustment for household clustering.
Turner *et al*. (1993) [23]	CS	Tanzania (Kongwa district, Dodoma region)	4,932 women, 18–60 years.	Sleeping in a room with cooking fire during childbearing years.	Trichiasis: 1.8 (1.2–2.8)	Age, mother had trichiasis, no. of child deaths, never married, no adult education, wood/earth home.	Large sample; clinical confirmation.	Fuel type, stove not described. We can infer from the area in Tanzania that this was a straight fire, using wood or charcoal.
Mesfin *et al*. (2006) [58]	CS	Ethiopia (Tigray)	3,900 people from 1,200 households.	No chimney in kitchen.	Trichomatous follicles/ Inflammation: 0.91 (0.84–1.61) Trichomatous scarring: 1.22 (1.04–1.54) Trichomatous trichiasis: 1.25 (1.03–1.89)	Sex, age, education, urban/rural residence, latrine, waste disposal, no. days since washed face, use of soap to wash face, time spent fetching water.	Large sample; clinical confirmation.	Fuel type, stove not described.
*Outcome: Blindness and visual impairment*								
Mishra *et al*. (1999) [59]	CS	India	173,520 people, >29 years (from 1992–1993 National Family Health Survey).	Use of biomass fuels *vs*. other fuels (gas, coal, kerosene, electricity).	Partial or complete blindness: 1.32 (1.16–1.50)	Separate kitchen, house type, crowding, age, gender, urban/ rural residence, education, religion, case/tribe, geographic region.	Very large, population-based sample.	No clinical confirmation. Cause of blindness unknown. Mixing of fuel types in reference category.
Freeman *et al*. (2010) [60]	CS	Burkina Faso	4,822 people, both sexes.	Cooking stove located in kitchen area.	Near visual difficulty: 1.43 (1.01–2.02) Far visual difficulty: 1.13 (0.81–1.59)	Age, sex, education, fruit consumption, vegetable consumption, current smoking.	Large population.	Self report—no clinical confirmation. Cause of visual difficulty unknown. Fuel type, stove not described.
*Outcome: Tears while cooking*								
Khushk *et al*. (2005) [18]	CS (following an intervention)	Pakistan	45 women with chimney stoves; 114 women using traditional stoves.	Stove type.	0.54 (0.22–1.30)	Age, education, husband/father’s education, house construction, income, electric connection in household, smokers in house.		Small sample, Self reported symptoms. No clinical eye exams.
*Outcome: Sore eyes in past month*								
Díaz *et al*. (2007) [16]	RT	Guatemala	259 women with improved stoves (planchas); 245 with open fires.	Randomized assignment.	For open fires relative to planchas: 5.56 (3.45–9.09)		Only RT to investigate stoves	Self-reported symptoms only. No clinical eye examination.

^1^ CC: Case-control study; CS: Cross-sectional; RT: Randomized trial. ^2^ No. cases/No. controls for case-control studies. ^3^C cortical, N nuclear, M mixed, P posterior subcapsular.

The tear film, which protects the ocular surface, is a complex, multilayered fluid that is exposed directly to the air and hence affected by air quality, as has been demonstrated in many studies and summarized by Wolkoff [61]. It can be altered by physical processes that increase tear evaporation and/or decrease tear film stability (e.g., heat, blowing air, humidity), as well as by aerosols and combustion products that alter its biochemical composition and structure. For example, HAP components such as formaldehyde, acrolein, and particulate matter could induce oxidative stress and alter the cytokine content of tears and ocular surface, leading to inflammation and development of dry eye disease [62,63,64,65,66]. Moreover, exposure to ambient pollution, nitrogen dioxide, formaldehyde, second hand tobacco smoke and wood smoke have all been associated with symptoms of ocular discomfort and/or the development of tear film instability [67,68,69,70,71,72,73,74]. Changes in the ocular surface environment associated with cigarette smoking include reductions in tear break-up time, changes in the tear film lipid layer, reductions in tear secretion, corneal and conjunctival sensitivity, and tear lysozyme concentration [14,75,76]. Recently, secondhand tobacco smoke exposure was associated with similar effects [77]. On the other hand, exposure to indoor airborne dust has shown inconsistent findings with regard to decreased tear break up time and dry eye symptoms [78,79], although it is possible that the constituents of the dust may be a factor [79]. Of interest, there are suggestive data that increases in ambient levels of particulate matter and carbon monoxide in air pollution may be associated with meibomian gland dysfunction—a common eyelid disease [80]. While we could find no studies explicitly linking HAP to ocular surface changes or dry eye disease, there is biological plausibility for such an association and it warrants further research.

### 3.2. Exacerbations of Infectious Eye Diseases

#### 3.2.1. Trachoma/Trichiasis

Infectious eye diseases are very common in resource-limited countries. While the diseases themselves are caused by infectious agents, there are data to suggest that the manifestations may be exacerbated by HAP. Trachoma, caused by repeated re-infections with *C. trachomatis*, is the leading infectious cause of blindness, and is endemic in parts of Asia, the Middle East, Latin America, and sub-Saharan Africa [81]. In trachoma-endemic communities, young children have active, follicular disease and are the reservoirs of infection. Women are four times more likely than men to have blinding sequelae of trachoma, scarring and trichiasis. There is biological plausibility for an association with HAP: trachoma and, in particular, trachomatous scarring, are immunopathological responses to infection. Chronic smoke irritation of the conjunctival surface could exacerbate the inflammatory response and promote conjunctival scarring, which can lead to trichiasis. The ocular surface irritation could also cause people to rub their eyes more frequently, increasing the risk of transmission of *Chlamydia trachomatis*.

Four cross-sectional studies have reported increased risks of trachoma or trichiasis in relation to biomass use-related parameters [22,23,57,58]. However, the studies used indirect measures of exposure, including having a cooking fire in the sleeping room, cooking in a central room *vs*. cooking in a separate room, and having no chimney in the kitchen. Three studies produced some evidence of an increased risk and one a protective effect of HAP exposure. Overall, the weight of evidence suggests that use of indoor cooking fires and cookstoves may increase risk of active trachoma, and possibly trichiasis. To date, scarring trachoma has not been evaluated for an association with HAP. 

#### 3.2.2. Corneal Infections

Corneal ulcers are more common in developing countries, and are often related to infections following corneal abrasions during agricultural work [82]. We did not identify any articles related to corneal ulcers or wound healing in association with HAP. As noted, with the possible adverse effects of HAP on the ocular surface, there may be a risk of corneal ulcers from delayed wound healing after infection. This association might be difficult to study retrospectively as many of the eyes with injuries or infections in developing countries are lost due to delayed or improper care [83].

## 4. Discussion

With the exception of cataract, there have been few studies to date on the possible associations between HAP and risk of eye disease, although there are biological reasons to hypothesize linkages. For cataract, the evidence indicates that there is an association with household biomass fuel use. Limitations in study design and reporting restrict definitive conclusions about the magnitude of the association. A well-conducted population-based study to more accurately define the relationship would therefore be advantageous for cost-benefit analysis and clarification of the contribution of HAP to the global burden of cataract. Quantification of lens opacity in relation to the degree of smoke exposure should be considered, as this may provide evidence of exposure-response relationships and is likely to be more sensitive to the risk with individual cataract subtypes, as shown in a recent study in Nepal [32].

The limited evidence for active trachoma in children and trichiasis in women suggests associations with household biomass fuel use, although the assessment tools were fairly crude. Prospective studies in children living in communities with active trachoma could readily be carried out. Trachomatous scarring could also be assessed because incidence and progression generally occur in a relatively short time window [84]. Randomized stove intervention trials in areas hyperendemic for trachoma should include investigation of active trachoma in children (the reservoirs of infection and acute trachoma) to investigate whether an association with household smoke exposure exists. In addition, studies of trachoma in clinical trials could easily add a risk factor assessment that includes exposure to HAP. If confirmed, this association would support expanding partnerships with the neglected tropical disease community, and advocacy for interventions to reduce HAP. For example, the provision of more efficient and less-polluting cookstoves and/or cleaner fuels can be one of the environmental improvements for trachoma control. Longer term studies in trachoma-endemic areas should consider inclusion of assessment of the later stages of trachoma, particularly scarring trachoma.

The limited available evidence suggests that smoke reduction from cleaner cookstoves or cleaner fuel reduces ocular surface irritation. This evidence could be confirmed and strengthened by the inclusion of simple questions about ocular symptoms in other ongoing or planned intervention studies of cleaner burning stoves. The possibility of using tears and conjunctival cells as biological media for the investigation of biomarkers of HAP exposure also holds promise. Moreover, the visual impact of HAP due to ocular irritation and/or dry eye disease could be investigated with questions about visual symptoms, such as:

- How often do you experience blurred vision? (constantly, often, sometimes, never)
- How often do you experience fluctuating vision? (constantly, often, sometimes, never)
- How often do problems with your vision affect your ability to perform your usual household activities (e.g., cooking)? (constantly, often, sometimes, never)

There is the possibility that eye diseases are related more broadly to indoor air pollution, particularly from industrial indoor air pollution. This was not addressed in this article, because we chose to focus on the excess risk of blindness, and causes of blindness, in women in developing countries, and it is likely that HAP may be a larger contributor to this gender imbalance.

As far as practicable, the following principles should guide the design and conduct of future epidemiologic studies investigating the link between HAP and ocular disorders. First and foremost, it must be acknowledged that HAP is a term that covers a multiplicity of exposures and not all exposures may be causative. In this section, the recommendations are for studies that at least establish a link with the eye disease of interest, and if such an association is found, further detailed work on compounds of interest, possible related mechanisms, and pathways is justified. The following considerations for the epidemiological studies are important:

(a) Adequate sample size, based on appropriate power calculations.
(b) For case-control studies, careful attention to selection of controls, so that the controls are representative of the base population that gave rise to the cases. Preferably, studies should be population-based, as the possibility of control selection bias is difficult to eliminate from hospital- or clinic-based studies.
(c) In hospital- or clinic-based case-control studies, documentation of control diagnoses.
(d) Collection of histories of stove and fuel type use, and kitchen ventilation.
(e) Collection of data on a comprehensive range of covariates/potential confounders, including household lighting type and any use of fuels for heating.
(f) Micro-environment monitoring for particulates and carbon monoxide in a sample of kitchen environments and, if possible, personal monitoring of a sample of study participants for the same pollutants. However, lack of facilities for monitoring or the cost of such monitors may hinder the undertaking of such studies. Simple questions on stove and fuel type have been widely used and have proven very effective in identifying health outcomes associated with HAP.
(g) Separate treatment of fuel/stove types in the statistical analysis.

## 5. Conclusions

Limited direct evidence exists and no firm conclusions can be drawn about associations with household solid fuel use and major blinding eye diseases, although the strongest evidence is for an association with cataract. Given the high burden associated with these conditions, the widespread use of solid fuels for cooking, and the plausibility of associations, appropriate investigations are needed.
